# Characterisation of transgenic pigs expressing a human T cell‐depleting anti‐CD2 monoclonal antibody

**DOI:** 10.1111/xen.12836

**Published:** 2023-11-13

**Authors:** Evelyn J. Salvaris, Nella Fisicaro, Stephen McIlfatrick, Adwin Thomas, Erin Fuller, Andrew M. Lew, Mark B. Nottle, Wayne J. Hawthorne, Peter J. Cowan

**Affiliations:** ^1^ Immunology Research Centre St. Vincent's Hospital Melbourne Fitzroy Victoria Australia; ^2^ Robinson Research Institute and School of Biomedicine University of Adelaide Adelaide South Australia Australia; ^3^ The Centre for Transplant & Renal Research Westmead Institute for Medical Research Westmead New South Wales Australia; ^4^ Walter and Eliza Hall Institute Department of Medical Biology and Department of Microbiology & Immunology University of Melbourne Melbourne Victoria Australia; ^5^ Department of Surgery Westmead Hospital School of Medical Sciences University of Sydney Westmead New South Wales Australia; ^6^ Department of Medicine University of Melbourne Melbourne Victoria Australia

**Keywords:** anti‐CD2 monoclonal antibody, diliximab, neonatal islet cell clusters, porcine, xenotransplantation

## Abstract

**Background:**

Pig islet xenotransplantation is a potential treatment for type 1 diabetes. We have shown that maintenance immunosuppression is required to protect genetically modified (GM) porcine islet xenografts from T cell‐mediated rejection in baboons. Local expression of a depleting anti‐CD2 monoclonal antibody (mAb) by the xenograft may provide an alternative solution. We have previously reported the generation of *GGTA1* knock‐in transgenic pigs expressing the chimeric anti‐CD2 mAb diliximab under an MHC class I promoter (MHCIP). In this study, we generated *GGTA1* knock‐in pigs in which MHCIP was replaced by the β‐cell‐specific porcine insulin promoter (PIP), and compared the pattern of diliximab expression in the two lines.

**Methods:**

A PIP‐diliximab knock‐in construct was prepared and validated by transfection of NIT‐1 mouse insulinoma cells. The construct was knocked into *GGTA1* in wild type (WT) porcine fetal fibroblasts using CRISPR, and knock‐in cells were used to generate pigs by somatic cell nuclear transfer (SCNT). Expression of the transgene in MHCIP‐diliximab and PIP‐diliximab knock‐in pigs was characterised at the mRNA and protein levels using RT‐qPCR, flow cytometry, ELISA and immunohistochemistry. Islets from MHCIP‐diliximab and control *GGTA1* KO neonatal pigs were transplanted under the kidney capsule of streptozotocin‐diabetic SCID mice.

**Results:**

NIT‐1 cells stably transfected with the PIP‐diliximab knock‐in construct secreted diliximab into the culture supernatant, confirming correct expression and processing of the mAb in β cells. PIP‐diliximab knock‐in pigs showed a precise integration of the transgene within *GGTA1*. Diliximab mRNA was detected in all tissues tested (spleen, kidney, heart, liver, lung, pancreas) in MHCIP‐diliximab pigs, but was not detectable in PIP‐diliximab pigs. Likewise, diliximab was present in the serum of MHCIP‐diliximab pigs, at a mean concentration of 1.8 μg/mL, but was not detected in PIP‐diliximab pig serum. An immunohistochemical survey revealed staining for diliximab in all organs of MHCIP‐diliximab pigs but not of PIP‐diliximab pigs. Whole genome sequencing (WGS) of a PIP‐diliximab pig identified a missense mutation in the coding region for the dixilimab light chain. This mutation was also found to be present in the fibroblast knock‐in clone used to generate the PIP‐diliximab pigs. Islet xenografts from neonatal MHCIP‐diliximab pigs restored normoglycemia in diabetic immunodeficient mice, indicating no overt effect of the transgene on islet function, and demonstrated expression of diliximab in situ.

**Conclusion:**

Diliximab was widely expressed in MHCIP‐diliximab pigs, including in islets, consistent with the endogenous expression pattern of MHC class I. Further investigation is required to determine whether the level of expression in islets from the MHCIP‐diliximab pigs is sufficient to prevent T cell‐mediated islet xenograft rejection. The unexpected absence of diliximab expression in the islets of PIP‐diliximab pigs was probably due to a mutation in the transgene arising during the generation of the knock‐in cells used for SCNT.

**Abbreviations**
αGal: galactose‐α1,3‐galactose
GM: genetically modified
GTKO: α1, 3‐galactosyltransferase gene‐knockout
IPGTT: intraperitoneal glucose tolerance test
mAb: monoclonal antibody
MFI: mean fluorescence intensity
MHCIP: MHC class I promoter
NICC: neonatal islet cell cluster
PIP: pig insulin promoter
RT‐qPCR: reverse transcription–quantitative polymerase chain reaction
SCNT: somatic cell nuclear transfer
WT: wild type

## 1 INTRODUCTION

Globally, approximately 22 000 000 people suffer from type 1 diabetes,^1^ an autoimmune disease that destroys the β cells that produce insulin for regulation of blood glucose. Insulin therapy is improving, but does not fully restore physiological blood glucose control. Transplantation is the best treatment option, but is limited by the shortage of suitable human donor organs. The promising results from the first clinical pig‐to‐human heart transplant,^2^ along with several experimental pig‐to‐deceased human transplants of kidneys,^3^, ^4^ highlight the prospect of using genetically modified (GM) pig organs to treat end‐stage diseases. However, in the case of type 1 diabetes, the benefits of restoring normoglycemia by xenotransplantation of pig islets must be balanced against the risks and side effects of the ongoing immunosuppression required to prevent rejection. To address this issue, we have developed a model of “local” immunosuppression (i.e., secretion of immunomodulators at the transplant site by the xenograft).^5^, ^6^

We recently reported the long‐term survival and function of GM pig neonatal islet cell clusters (NICCs) in immunosuppressed diabetic baboons.^7^ The donors expressed the human complement regulators CD55 and CD59 and a human glycosyltransferase, and were knocked out for the *GGTA1* gene, which is responsible for synthesis of the major carbohydrate xenoantigen αGal. Immunosuppression consisted of induction with the non‐activating chimeric anti‐CD2 mAb diliximab to deplete T cells and NK cells, a short course of tacrolimus, and maintenance with the co‐stimulation blockade agents belatacept and anti‐CD154. Diliximab was chosen because we have shown it depletes both human and baboon T cells in vivo, and blocks human T cell activation via the CD2‐CD58 co‐stimulation pathway.^5^, ^8^ Insulin independence was achieved for a mean of nearly 400 days, with a maximum of 675 days. When maintenance immunosuppression was electively ceased, recipients eventually lost xenograft function and showed evidence of T cell‐mediated rejection.^7^

We postulate that genetically mediated local secretion of diliximab by the islets may eliminate infiltrating T cells after the cessation of immunosuppression and prolong xenograft function indefinitely, without systemic side effects. To investigate this, we generated knock‐in pigs containing a diliximab transgene integrated into (and inactivating) *GGTA1*.^6^ Expression of diliximab was regulated by an MHC class I promoter (MHCIP), the mouse H‐2K^b^ promoter, which we had earlier shown to drive widespread transgene expression in pig tissues including islets.^9^, ^10^, ^11^ Diliximab was detected in the serum of the MHCIP‐diliximab knock‐in pigs using a FACS‐based assay. Importantly the pigs were not immunocompromised, as diliximab does not deplete porcine T cells.^6^

Whether the level of MHCIP‐driven diliximab expression in porcine islets will be sufficient to protect NICC xenografts remains to be determined, leading us to explore the use of a different promoter. Our earlier in vitro studies demonstrated that reporter gene expression in transduced pig NICCs was higher using the β‐cell‐specific rat insulin promoter compared to MHCIP.^12^ Furthermore, Klymiuk et al reported that the pig insulin promoter (PIP) drove strong β‐cell‐specific expression of another secreted immunomodulatory molecule, LEA29Y (belatacept), in transgenic pigs.^13^ The aims of the current study were therefore to (i) generate PIP‐diliximab *GGTA1* knock‐in pigs, and (ii) evaluate and compare the pattern and level of diliximab expression in the MHCIP‐diliximab and PIP‐diliximab knock‐in pigs, with particular regard to pancreatic islets.

## 2 MATERIALS AND METHODS

### 2.1 Animals and ethical approval

All experiments using animals and human samples were performed in accordance with the relevant guidelines and regulations. Genetically modified pigs were generated and used with the approval of the Animal Ethics Committee of the University of Adelaide (approval number M‐2021‐08B) in accordance with the Australian Code of Practice for the Care and Use of Animals for Scientific Purposes (National Health and Medical Research Council, 2013). Human blood samples were collected from consenting healthy volunteers with approval from the St Vincent's Hospital Melbourne Human Research Ethics Committee (approval number HREC 098/18).

The generation of GGTA‐1 knockout and MHCIP‐diliximab knock‐in pigs has been described previously.^6^, ^14^

### 2.2 Generation and validation of PIP‐diliximab knock‐in construct

**FIGURE 1:**
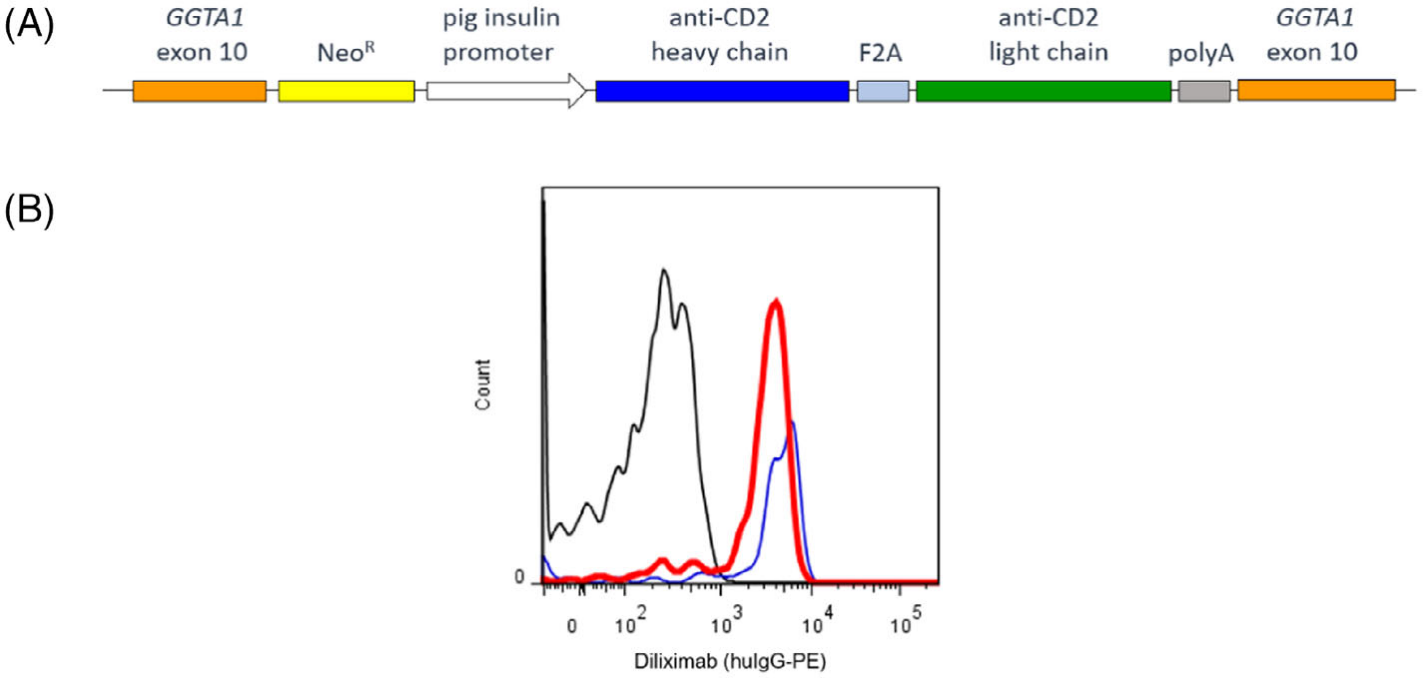
Validation of the PIP‐diliximab *GGTA1* targeting construct. (A) Schematic of the construct (not to scale) showing the 5′ and 3′ homology arms of *GGTA1* exon 10, which encodes the catalytic domain of α1,3‐galactosyltransferase; neomycin resistance gene (Neo^R^); 1.3‐kb regulatory sequence from the pig insulin promoter; coding regions of the heavy and light chains of diliximab linked by a furin‐2A signal; and polyadenylation signal. (B) Detection of diliximab produced by stably transfected mouse NIT‐1 insulinoma cells. Culture media from PIP‐diliximab‐transfected (red line) or untransfected (black line) cells, or purified diliximab (blue line), were incubated with human leukocytes. Diliximab binding to CD3+ T cells was detected using a PE‐conjugated anti‐human IgG.

The MHC‐I promoter was excised from the MHCIP‐diliximab *GGTA1* knock‐in construct^6^ and replaced with the pig insulin promoter including the first exon and intron.^13^ The resulting PIP‐diliximab knock‐in construct (Figure 1A) was stably transfected into the mouse insulinoma cell line NIT‐1 with neomycin selection. Culture media from confluent pooled neomycin‐resistant stable transfectants were screened for the presence of diliximab by flow cytometry of human T cells as described.^6^

### 2.3 Generation and genotyping of PIP‐diliximab knock‐in pigs

WT pig fetal fibroblasts were co‐transfected by nucleofection with the PIP knock‐in construct and expression vectors for the high‐fidelity Cas9 variant *Fok*I‐dCas9 and the *GGTA1* exon 10 guide RNAs GT3 and GT4 as described.^6^ [Note that the target for these gRNAs was originally defined as exon 9,^6^ but an additional exon in *GGTA1* has since been identified.] Neomycin‐resistant clones were screened by flow cytometry for cell surface expression of αGal using IB4‐FITC (Sigma‐Aldrich, St Louis, MO). αGal‐negative clones were screened by PCR for correct integration of the transgene and absence of vector integration as described.^6^ Piglets were generated by somatic cell nuclear transfer and genotyped by PCR as described.^6^

### 2.4 Detection of diliximab in media and sera by flow cytometry and ELISA

The concentration of diliximab in culture media and porcine sera was initially estimated by flow cytometry of human T cells as described.^6^ For a more sensitive and quantitative assessment, a diliximab ELISA was developed. 96‐well Nunc MaxiSorb microtitre plates (ThermoFisher Scientific, Scoresby, Vic, Australia) were coated with 10 μg goat anti‐human IgG (eBiosciences, San Diego, CA), blocked in 5% Blotto in PBS/0.05% Tween 20 for 3 h at 37°C, and incubated with sample for 1 h at 37°C. Bound diliximab was detected by incubating with peroxidase‐conjugated mouse anti‐human IgG3‐HRP (Southern Biotech, Birmingham, Al) for 1 h at 37°C followed by Sure Blue TMB peroxidase substrate (KPL, Gaithersburg, MD) for 30 min at room temperature. Absorbance at 450 nm was read on a FLUOStar Omega plate reader (BMG Labtech, Offenburg, Germany). Purified diliximab (0.012–62.5 ng/mL; GenScript, Piscataway, NJ) was used to generate a standard curve.

### 2.5 RT‐qPCR

Tissue samples were stored in RNAlater (Invitrogen, Waltham, MA) for 24–48 h and then at −80°C until RNA isolation. Total RNA was extracted from 20 mg of tissue using ReliaPrep RNA Tissue Mini Prep Systems (Promega Australia, Alexandria, NSW, Australia), and quantitated using a FLUOstar Omega plate reader. 2.0 μg of total RNA was treated with Turbo DNase (Invitrogen, Waltham, MA) for 1 h at 37°C followed by DNase inactivation. One μg of oligo(dT) and 2 μg of random primers (Invitrogen, Waltham, MA) were annealed to DNAse‐treated RNA at 70°C for 10 min in 50 μL reaction volume. First‐strand cDNA synthesis was performed using 25 μL primer‐annealed RNA, 10 nM dNTPs, 60 U RNAseOUT recombinant RNase inhibitor, 0.1 M DTT and 5x first‐stand buffer (Invitrogen, Waltham, MA) in a 100 μL final volume with or without 300 U Superscript III (Invitrogen, Waltham, MA,USA) at 50°C for 45 min, then 70°C for 10 min. cDNA was diluted 1/10 and reverse transcriptase quantitative PCR was performed using 1x TaqMan Gene expression master mix and custom TaqMan gene expression assay for diliximab (Forward primer 5′‐GTGCTGATTCTTTTGTGG‐3′, Reverse primer 3′‐TTGAGAGATGGGTTGTAG‐3′, probe AAACTGGAGTGGATGGGCT‐FAM MGB) and TaqMan β actin (ACTB: Ss03376081_u1) using a 7500Fast Real‐Time PCR system (Applied Biosystems, Waltham, MA). Diliximab mRNA levels were normalized to the expression of the porcine β actin gene.

### 2.6 Isolation of porcine neonatal islet cell clusters (NICCs)

NICCs were isolated from neonatal pigs as previously described.^7^, ^11^ In brief, pancreata were dissected and digested with 2.5 mg/mL Collagenase Type V (Sigma‐Aldrich, St Louis, MO) at 37°C. Cell suspensions were washed to remove debris and cultured in Hams F‐10 containing 1% pig serum for 6 days, with media changed and sampled every second day. NICCs were pooled from culture and counted prior to transplantation.

### 2.7 Transplantation of porcine NICCs into diabetic SCID mice

SCID mice were injected with 180 mg/kg streptozotocin to induce diabetes with fasting blood sugar levels (BSL) greater than 15 mmol/L. Diabetic mice were transplanted under the left renal capsule with up to 4,500 MHCIP‐diliximab knock‐in or GTKO NICCs/mouse. BSL were measured daily for up to 90 days, with insulin administered for BSL above 12 mmol/L. An intraperitoneal glucose tolerance test (IPGTT) was performed prior to nephrectomy.^15^

### 2.8 Immunohistochemistry

Pig tissues and NICC xenograft‐bearing mouse kidneys were embedded in OCT. 5 μM tissue sections were fixed with 5% PFA, blocked with 1%–3% H_2_O_2_, avidin/biotin blocking kit (Vector Laboratories, Burlingame, CA) and 10% goat serum, and incubated with goat anti‐human IgG‐biotin (eBiosciences, San Diego, CA) followed by streptavidin‐HRP (Agilent Technologies Australia, Mulgrave, Vic, Australia) and detected using liquid DAB. Guinea pig anti‐insulin and donkey anti‐guinea pig IgG‐HRP were used to stain for insulin on pig pancreas. Sections were counterstained with hematoxylin, mounted in organol limonene (Sigma‐Aldrich, St Louis, MO), coverslipped and viewed using bright field microscopy.

### 2.9 Whole Genome Sequencing (WGS)

Genomic DNA was purified from PIP‐diliximab pig tissue using High Pure PCR Template Preparation Kit (Roche). WGS and bioinformatics analysis were performed by AGRF Ltd (Melbourne, Australia).

## 3 RESULTS

### 3.1 Generation of porcine insulin promoter‐diliximab (PIP‐diliximab) knock‐in pigs

**FIGURE 2:**
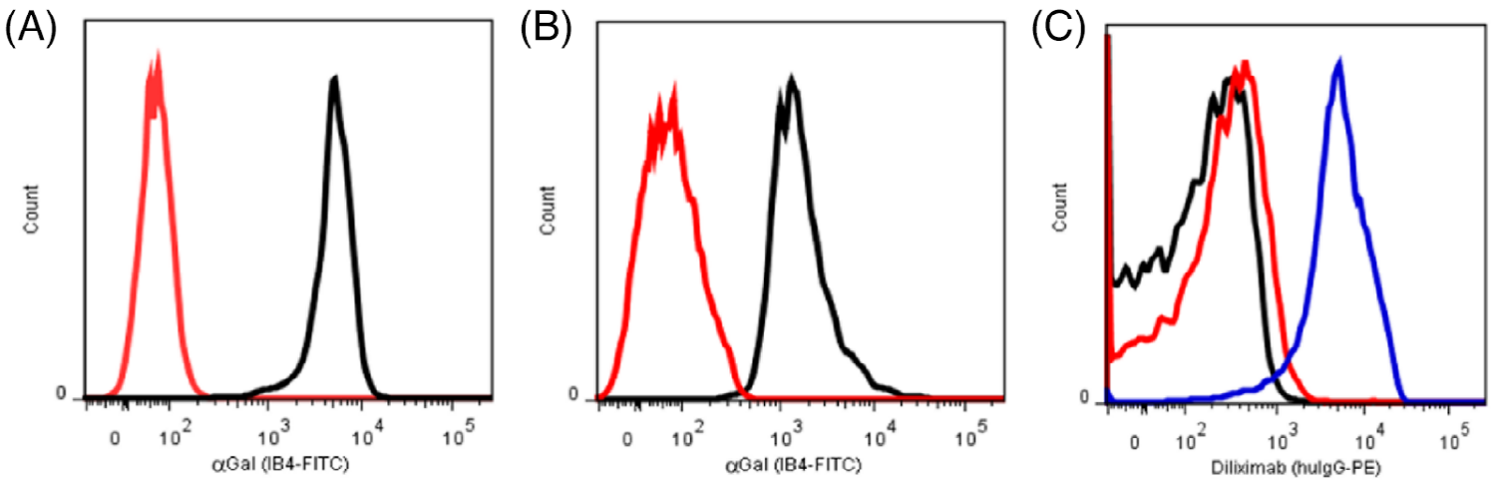
Flow cytometric analysis of αGal and diliximab expression in pig fetal fibroblasts and cloned pigs. (A) Absence of αGal expression in a fibroblast clone stably transfected with the PIP‐diliximab GGTA1 knock‐in construct (red line = clone, black line = non‐transfected WT pig fibroblasts). (B) Absence of αGal expression in peripheral blood leukocytes from a PIP‐diliximab knock‐in pig (red line = knock‐in, black line = WT). (C) Expression of diliximab in serum from MHCIP‐diliximab knock‐in pig (blue line) but not PIP‐diliximab knock‐in (red line) or non‐transgenic (black line) pigs. 5000 cells per marker were counted.

The 7.1 kb PIP‐diliximab knock‐in construct (Figure 1A, not to scale) was validated by transfection into the NIT‐1 mouse insulinoma cell line. Pooled stable transfectants contained diliximab in the culture medium (Figure 1B), confirming correct expression and secretion of the mAb by β cells in vitro. The knock‐in construct was stably co‐transfected with expression vectors for *Fok*I‐dCas9 and *GGTA1* exon 10 guide RNAs into male WT pig fetal fibroblasts. 22 of 152 neomycin‐resistant clones were expanded, and 3/22 clones screened negative for αGal by flow cytometry (Figure 2A). PCR and sequencing of the three clones confirmed precise integration of the PIP‐diliximab transgene into exon 10 of *GGTA1* (Figures S1A–C), and absence of genomic integration of the expression vectors (Figure S1D). All three clones contained an identical 26 bp deletion of GTGCCTTGTACCACCAGGCCTGTAGC in the target region of the second *GGTA1* allele. Diliximab was not detected in the culture supernatant of the clones (data not shown), but this was not unexpected given the β‐cell specificity of the pig insulin promoter.

One clone was used to produce pigs by somatic cell nuclear transfer. One hundred thirty‐four cloned blastocysts were transferred into eight foster sows, resulting in three pregnancies and eight live born pigs. All pigs were transgenic for the diliximab construct (Figure S2), and flow cytometric analysis of peripheral blood leukocytes from pigs up to 13 months old confirmed the absence of αGal expression (Figure 2B). All pigs were healthy, developed normally and had a normal full blood count (Table S1).

### 3.2 Serum levels of diliximab in MHCIP‐diliximab and PIP‐diliximab knock‐in pigs

Using a semi‐quantitative flow cytometric method based on staining of human T cells^6^ we detected the presence of diliximab in serum from MHCIP‐diliximab knock‐in pigs, but not in serum from PIP‐diliximab knock‐in pigs (Figure 2C). This is consistent with the expected broad transgene expression from MHCIP versus highly restricted expression from PIP. We next established a quantitative and sensitive ELISA using purified diliximab as standard. The mean serum concentration of diliximab was 1.8 μg/mL in MHCIP‐diliximab knock‐in pigs, whereas the mAb was undetectable in PIP‐diliximab knock‐in and non‐transgenic (GTKO) pig serum.

### 3.3 Tissue expression of diliximab mRNA in knock‐in pigs

**FIGURE 3:**
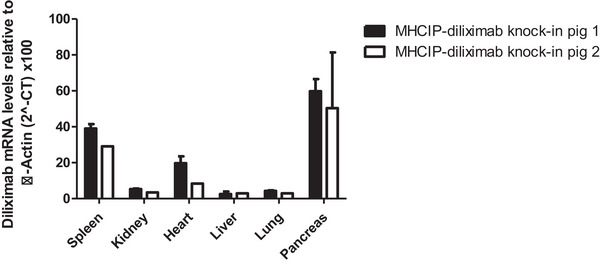
Diliximab transgene expression levels in organs of MHCIP‐diliximab knock‐in pigs. mRNA expression was measured by RT‐qPCR and expressed relative to pig β‐actin. Results are expressed as the mean of analysis of 2 samples from each organ, with the exception of kidney, spleen, heart, liver and lung (1 sample each) from pig 2. PIP‐diliximab knock‐in and non‐transgenic pigs did not express diliximab mRNA (data not shown).

Transgene expression at the mRNA level was determined by RT‐qPCR and calculated relative to the endogenous pig β actin gene, which has been demonstrated to be stably expressed under experimental conditions.^16^ Diliximab mRNA was detected in all MHCIP‐diliximab pig organs tested, with highest levels observed in the spleen and pancreas, followed by heart, kidney, liver and lung (Figure 3). Diliximab mRNA was not detected in any of these tissues in PIP‐diliximab pigs or non‐transgenic GTKO littermates (data not shown).

### 3.4 Immunohistochemical survey of diliximab expression in knock‐in pigs

**FIGURE 4:**
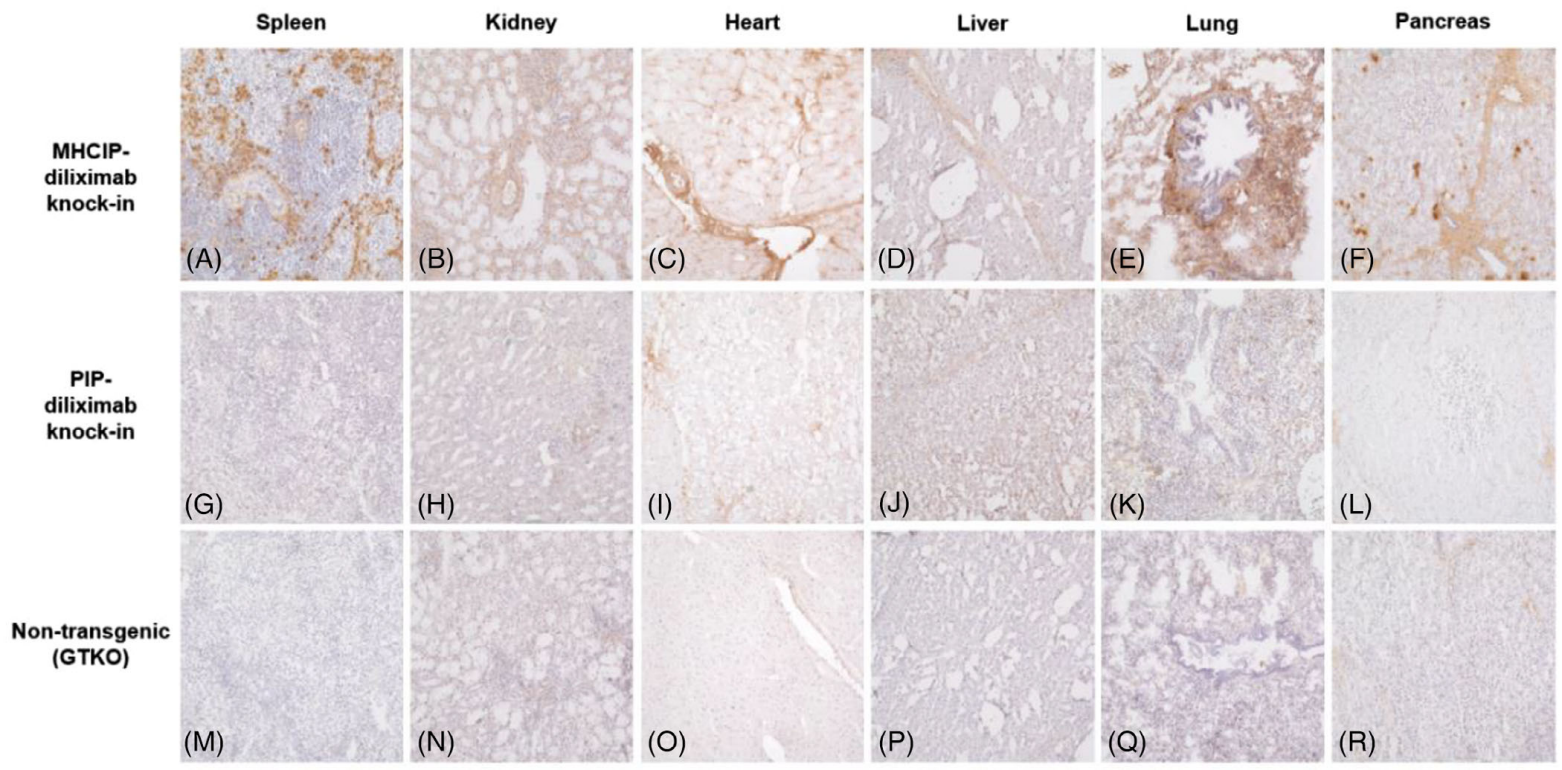
Immunohistochemical staining for diliximab in the organs of knock‐in pigs. In MHCIP‐diliximab knock‐in pigs, diliximab was expressed strongly in spleen (A) and lung (E), moderately in heart (C) and kidney (B), and weakly in liver (D). Strong but patchy expression was observed in pancreas (F). Diliximab expression was not detected in any organs of PIP‐diliximab knock‐in (G–L) or non‐transgenic GTKO (M–R) pigs. Magnification x20.

MHCIP‐diliximab pigs expressed diliximab at the protein level in all organs tested (Figure 4). Strong expression was observed in spleen (Figure 4A), lung (Figure 4E), and pancreas (Figure 4F), although not all cell types within these organs were positive. Expression in the heart was most evident on the vasculature (Figure 4C), while expression in the kidney (Figure 4B) and liver (Figure 4D) was relatively modest. Staining was absent in tissues from non‐transgenic GTKO pigs (Figure 4M–R).

**FIGURE 5:**
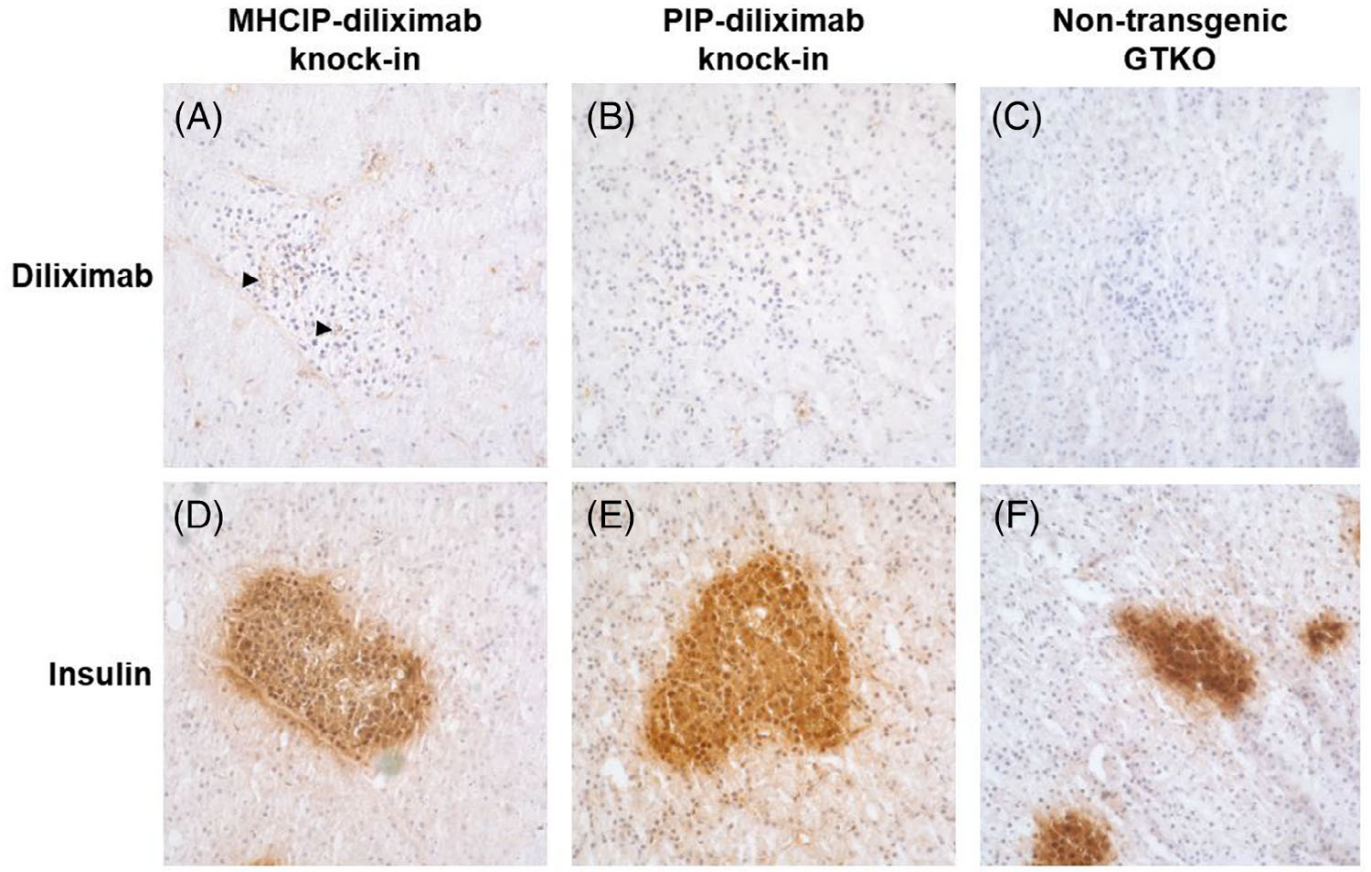
Immunohistochemical staining for diliximab (A–C) and insulin (D–F) in serial sections pf pancreas from MHCIP‐diliximab, PIP‐diliximab and non‐transgenic GTKO pigs. Weak expression of diliximab (arrowheads) was detected in islets from MHCIP‐diliximab knock‐in pigs (A), but was undetectable in PIP‐diliximab knock‐in (B) and non‐transgenic GTKO (C) islets. Magnification x40.

As expected, diliximab was not detectable in spleen, kidney, heart, liver or lung of PIP‐diliximab pigs (Figure 4G–K). Surprisingly, however, expression was also absent in pancreatic islets (Figure 4L). To investigate this further, pancreatic serial sections were stained for diliximab (Figure 5A–C) and insulin (Figure 5D–F). MHCIP‐diliximab islets showed weak expression of the mAb (Figure 5A), whereas PIP‐diliximab (Figure 5B) and control non‐transgenic GTKO (Figure 5C) islets were negative.

### 3.5 Whole genome sequencing (WGS)

To explore potential reasons for the failure of expression of the PIP‐diliximab transgene, WGS was performed on genomic DNA from a PIP‐diliximab knock‐in pig. WGS revealed that the transgene was correctly integrated in *GGTA1* and contained no structural rearrangements. However, a C→T mutation was identified in the diliximab coding region. PCR and sequencing confirmed that the mutation was present in the PIP‐diliximab pigs and further demonstrated its presence in the fibroblast clone used for SCNT to generate the pigs (Figure S3). It was not present in the transgene construct nor in two other knock‐in clones that were not used for SCNT (data not shown), indicating that the mutation arose randomly during the process of transfection and selection. This missense mutation is predicted to result in an amino acid change from threonine to isoleucine in the E strand (YSMSSTLTL) of the Ig light chain constant domain (Figure S4).

### 3.6 Transplantation of MHCIP‐diliximab NICCs into diabetic mice

To assess the function and diliximab secretion of neonatal islet cell clusters (NICCs) in a transplant setting, NICCs were isolated from MHCIP‐diliximab and control non‐transgenic GTKO pigs and transplanted under the kidney capsule of diabetic SCID mice. Several analyses were performed concurrently with transplantation. First, the transport media used to cold store MHCIP‐diliximab pancreata before NICC isolation was analysed by ELISA and found to contain a low level of diliximab (0.7 ng/mL), although this may have been due to contamination with residual blood rather than active expression by pancreatic cells. Diliximab was not present in the transport media of control pancreata. Second, the NICC culture media was also tested by ELISA. However, diliximab was not detected in the media of MHCIP‐diliximab NICCs. Possible reasons for the failure to detect diliximab may be weak transgene expression under these conditions and/or a dilution effect related to the large media‐to‐tissue volume ratio and frequent media changes. Finally, NICC samples were analysed for transgene mRNA expression by RT‐qPCR. Diliximab mRNA was present in MHCIP‐diliximab NICCs, at approximately 7.3% of the level in whole pancreas samples (Figure S5, Figure 3).

**FIGURE 6:**
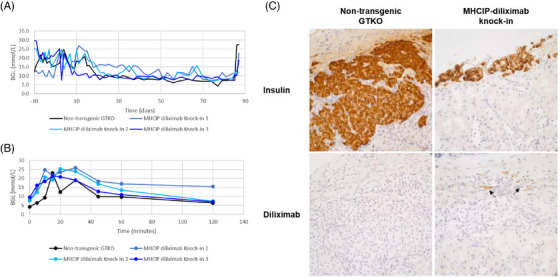
MHCIP‐diliximab NICC xenografts in mice demonstrate normal islet function and express diliximab. (A) Transplantation of MHCIP‐diliximab (*n* = 3) or non‐transgenic GTKO (*n* = 1) NICCs restored blood glucose control in diabetic SCID mice until removal of the graft‐bearing kidney on day 85. (B) IPGTT performed on day 77 post‐transplant. (C) Serial sections stained for insulin (i, ii) and diliximab (iii, iv), demonstrating weak expression of diliximab in MHCIP‐diliximab NICC xenografts (arrows, iv) but not in non‐transgenic GTKO xenografts (iii). Magnification x40.

Four diabetic SCID mice were transplanted with MHCIP‐diliximab (*n* = 3) or control non‐transgenic GTKO (*n* = 1) NICCs. The control transplant and two of the three MHCIP‐diliximab transplants gradually restored normoglycemia (Figure 6A) and a normal IPGTT response (Figure 6B) to the mice, indicating that transgene expression did not adversely affect islet function. Furthermore, immunohistochemical analysis demonstrated patchy diliximab staining in the vicinity of the MHCIP‐diliximab xenografts (Figure 6C), indicative of local expression.

## 4 DISCUSSION

In this study, we continued our investigation into the use of the anti‐CD2 mAb diliximab as a locally expressed immunomodulatory molecule to protect porcine xenografts from T cell‐mediated rejection. Anti‐CD2 is an attractive choice because it preferentially targets effector T cells while sparing regulatory T cells,^17^ and it can not only deplete but also inhibit co‐stimulation of T cells.^5^ Furthermore, we have achieved excellent results in the pig‐to‐baboon islet xenotransplantation model using diliximab as an induction agent.^7^

Examination of our existing line of MHCIP‐diliximab *GGTA1* knock‐in pigs revealed widespread tissue expression of diliximab, notably in spleen, lung and pancreas. Reflecting this, diliximab was readily detectable in serum. Immunohistochemical staining of the pancreas showed the presence of diliximab in islets, and analysis of NICCs before and after transplantation into diabetic mice confirmed transgene expression at the mRNA and protein level. Interestingly, mRNA and protein levels did not fully match in different organs (e.g., lung). The reason for this discrepancy is unclear, although the semi‐quantitative nature of immunohistochemistry makes it difficult to compare directly with RT‐qPCR; additionally, we cannot rule out sampling effects. The level of diliximab expressed by islets appeared to be considerably lower than that of other transgenes (human CD55 and CD59) that we have expressed in pigs using the same MHC class I promoter.^9^, ^11^ We suggest two possible reasons for this apparent discrepancy. First, cell membrane‐anchored proteins like CD55 and CD59 may be relatively easier to detect by immunohistochemistry than secreted molecules like diliximab, which disperse into surrounding tissue. Wijkstrom et al generated GM pigs expressing three transgenes (human TFPI, human CD39 and porcine CTLA4‐Ig) from the rat insulin promoter.^18^ Analysis of transgenic islets by immunofluorescence revealed stronger staining for the membrane‐bound TFPI than for the secreted CTLA4‐Ig.^18^ It should be noted, however, that Klymiuk et al demonstrated strong islet staining of another secreted protein (LEA29Y) in transgenic pigs from the pig insulin promoter.^13^

Second, the MHCIP‐CD55 and ‐CD59 pigs were produced by traditional transgenesis (oocyte microinjection), which results in integration of multiple transgene copies at random genomic sites and consequently, highly variable expression in different founder animals. This allowed us to select the highest expressing pig of the 16 transgenics generated^9^ to establish a line that was used for subsequent experiments.^7^, ^10^, ^11^ In contrast, the level of transgene expression in MHCIP‐diliximab knock‐in pig islets was essentially ‘pre‐determined’, not only by the strength of the promoter in β‐cells, but also by the transgene copy number (one) and the fixed site of integration (exon 10 of *GGTA1*). On the latter point, one important finding of our study is that the *GGTA1* integration site is permissive for transgene transcription in numerous cell types (Figure 4A–F), consistent with reports from other groups.^19^, ^20^

In an attempt to achieve stronger diliximab expression in islets, we generated a second line of *GGTA1* knock‐in pigs using the β‐cell‐specific porcine insulin promoter (PIP). We used the same 1.3‐kb promoter fragment reported to drive strong islet‐specific expression of LEA29Y in transgenic pigs.^13^ Surprisingly, diliximab was undetectable in the islets of PIP‐diliximab knock‐in pigs, despite the fact that the knock‐in construct had been validated in beta cells in vitro prior to generating the pigs. WGS of a PIP‐diliximab pig revealed a missense mutation predicted to cause a threonine to isoleucine change within the E strand of the Ig light chain constant domain of diliximab. We suggest that the substitution of a residue capable of hydrogen bonding (threonine) with one that is not (isoleucine) disrupted protein structure, causing degradation of and/or failure to detect misfolded diliximab. The absence of the mutation in other knock‐in clones that were not used to generate pigs highlights the need to sequence entire transgenes in clones destined for SCNT.

The level of diliximab expression in the MHCIP‐diliximab knock‐in pigs may prove adequate to protect xenografted solid organs such as the lung. However, the question remains whether the apparently weak expression in the islets of these pigs is sufficent to have a functional impact. In islet xenotransplantation, the balance of expression of secreted immunomodulatory molecules by xenografts – enough for protection, but not too much to cause systemic immunosuppression – will be key to the success of ‘local’ immunosuppression. If the level of the diliximab secreted by the islets is high enough to spill over into the circulation, the recipient may be at risk of developing malignancies or contracting infection or side effects similar to those associated with chronic immunosuppression therapies. For example, although NICC xenografts from PIP‐LEA29Y transgenic pigs strongly expressed the transgene^13^ and were protected from rejection for several months in a humanized mouse model,^21^ mice with established xenografts displayed a significant level of LEA29Y (mean 344 ng/mL) in their plasma. While we did not test NICCs from the MHCIP‐diliximab pigs in humanized mice, we have previously demonstrated proof of principle using transduced NICCs.^5^ In addition, a potential benefit of using an MHC class I promoter is inducibility by pro‐inflammatory cytokines.^22^ Even if basal expression of diliximab is insufficient to completely prevent T cell infiltration, it still may be efficacious as it may be able to block T cell activation in situ.^8^

## AUTHOR CONTRIBUTIONS

Evelyn J. Salvaris participated in the research design, performance of the research, data analysis, writing and critical revision of the manuscript. Nella Fisicaro assisted with the molecular and cell biology experiments. Stephen McIlfatrick performed somatic cell nuclear transfer experiments. Adwin Thomas, Erin Fuller, and Wayne J. Hawthorne performed the NICC isolations, Mouse transplant and IPGTT experiments. Andrew M. Lew, Mark B. Nottle, and Wayne J. Hawthorne participated in the concept and design of the study. Peter J. Cowan participated in the concept and design of the study, writing and critical revision of the manuscript, and carried the main responsibility for the study. All authors critically reviewed the manuscript.

## CONFLICT OF INTEREST STATEMENT

The authors of this manuscript declare that they have no conflict of interest to disclose.

## Supporting information

Supporting information

Supporting information

Supporting information

Supporting information

Supporting information

Supporting information
