# Supplementary material for: Characterisation of transgenic pigs expressing a human T cell‐depleting anti‐CD2 monoclonal antibody
Source: Xenotransplantation. 2023 Nov 13;31(1):e12836. doi: 10.1111/xen.12836 (PMC10909556; doi:10.1111/xen.12836)
Supplement: Supplementary file 1 — Supporting information [file XEN-31-e12836-s005.docx]

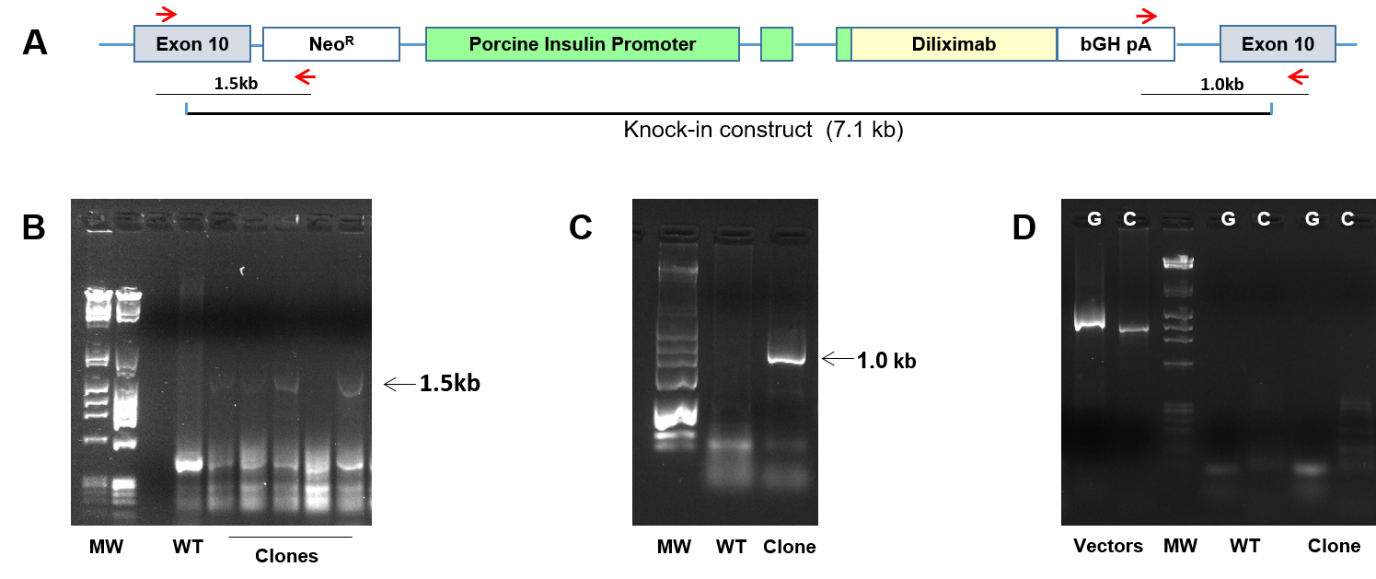


**Supplementary Figure 1.** PCR analysis to confirm correct integration of the PIP-diliximab knock-in construct and absence of CRISPR vectors in porcine fibroblast stable transfectant clones. (A) Schematic of the targeting construct showing the position of two PCR primer sets (arrows) that overlap the expected junctions of the *GGTA-1* integration site. (B) Amplification of the predicted 1.5 kb upstream junction PCR product in several stable clones. (C) Amplification of the predicted 1.0 kb downstream junction PCR product in the clone used for SCNT. (D) Left lanes: PCR products specific for the gRNA (G) and Cas9 (C) expression plasmids; right lanes: absence of these products in the clone used for SCNT. MW, molecular weight markers.
