# Supplementary material for: Characterisation of transgenic pigs expressing a human T cell‐depleting anti‐CD2 monoclonal antibody
Source: Xenotransplantation. 2023 Nov 13;31(1):e12836. doi: 10.1111/xen.12836 (PMC10909556; doi:10.1111/xen.12836)
Supplement: Supplementary file 2 — Supporting information [file XEN-31-e12836-s006.docx]

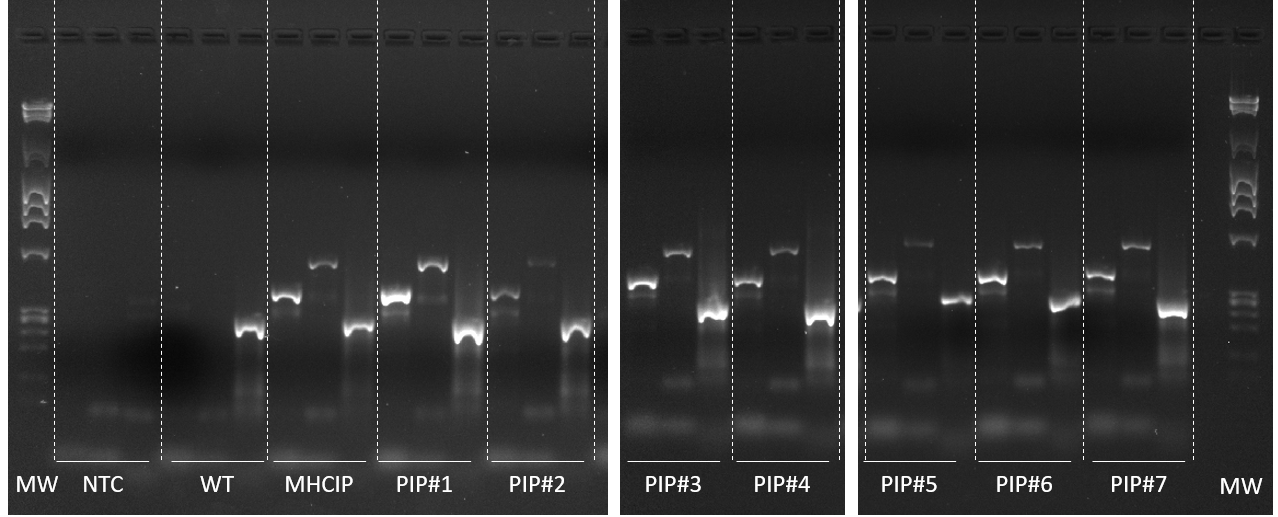


**Supplementary Figure 2.** PCR screening of PIP-diliximab knock-in piglets PIP#1-7 for the presence of the transgene. Three reactions were performed for each sample: diliximab heavy chain (408 bp product); diliximab light chain (550 bp product); and porcine 18S control (234 bp). MW, molecular weight markers; NTC, no template control; WT, WT pig negative control; MHCIP, MHCIP-diliximab pig positive control.
