# Supplementary material for: Characterisation of transgenic pigs expressing a human T cell‐depleting anti‐CD2 monoclonal antibody
Source: Xenotransplantation. 2023 Nov 13;31(1):e12836. doi: 10.1111/xen.12836 (PMC10909556; doi:10.1111/xen.12836)
Supplement: Supplementary file 3 — Supporting information [file XEN-31-e12836-s003.docx]

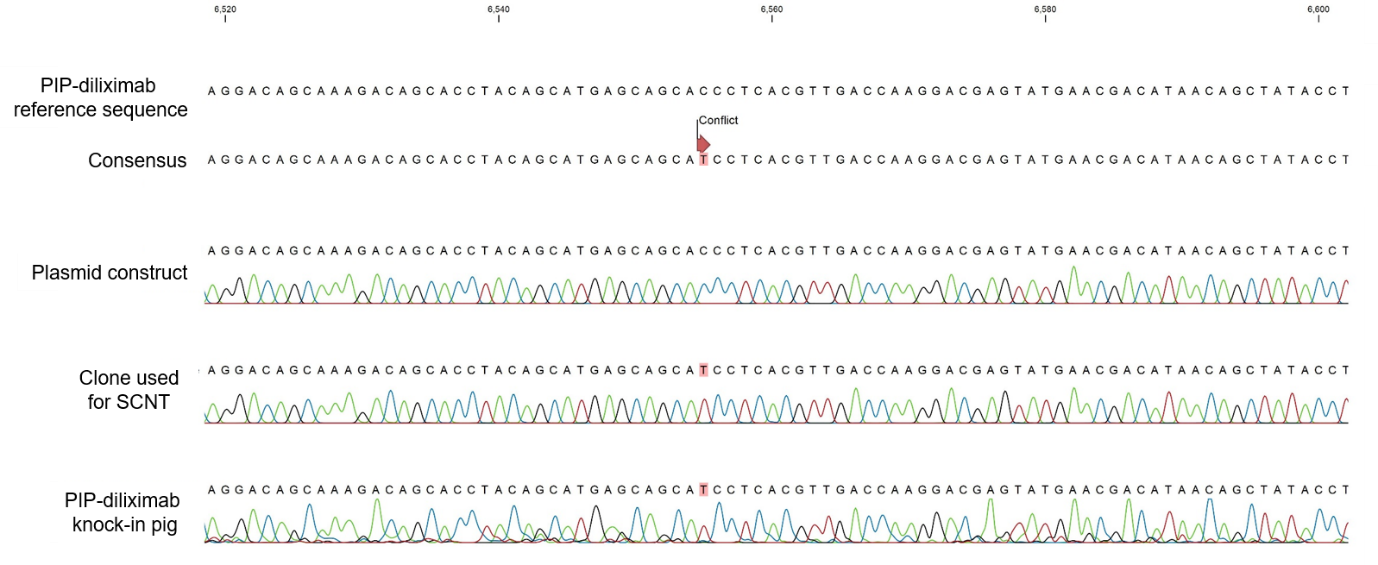


**Supplementary Figure 3.** Sequencing analysis of the diliximab light chain coding region. 1007 bp PCR products were amplified from genomic DNA isolated from the PIP-diliximab clone used for SCNT and a resulting PIP-diliximab knock-in pig, using primers TGATGACACAGTCTCCATCC (forward) and GGTGTTCCCCCAAAAATGGC (reverse). The PCR products were purified using PureLink Quick Gel Extraction Kit (Invitrogen, Waltham, MA) as per manufacturer’s instructions and sequenced (with the knock-in plasmid construct as control) by Sanger sequencing using BigDye Terminator v3.1 Cycling Sequencing Kit (Applied Biosystems, Waltham, MA, USA) as per manufacturer’s instructions. Sequence data were analysed using CLC Main Workbench 7.6.4 (Qiagen Australia, Clayton, Australia). Sequencing chromatograms above highlight the C to T mutation identified by WGS of a PIP-diliximab knock-in pig and demonstrate its presence in the fibroblast stable transfectant clone used for SCNT to generate the pig.
