# Supplementary material for: Characterisation of transgenic pigs expressing a human T cell‐depleting anti‐CD2 monoclonal antibody
Source: Xenotransplantation. 2023 Nov 13;31(1):e12836. doi: 10.1111/xen.12836 (PMC10909556; doi:10.1111/xen.12836)
Supplement: Supplementary file 4 — Supporting information [file XEN-31-e12836-s002.docx]

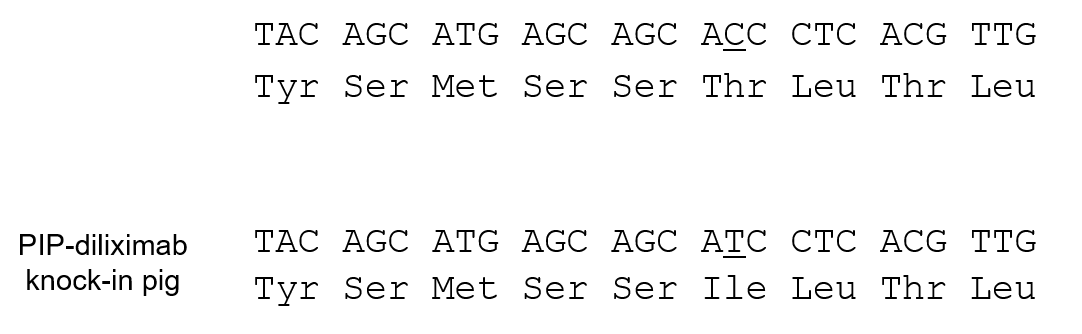


**Supplementary Figure 4.** Predicted amino acid change in the E strand of the Ig light chain constant domain of diliximab resulting from the missense mutation identified by WGS.
