# Supplementary material for: Characterisation of transgenic pigs expressing a human T cell‐depleting anti‐CD2 monoclonal antibody
Source: Xenotransplantation. 2023 Nov 13;31(1):e12836. doi: 10.1111/xen.12836 (PMC10909556; doi:10.1111/xen.12836)
Supplement: Supplementary file 5 — Supporting information [file XEN-31-e12836-s004.docx]

**Supplementary Figure 5.** MHCIP-diliximab knock-in NICCs express diliximab mRNA as determined by RT-qPCR.
