# Supplementary material for: Characterisation of transgenic pigs expressing a human T cell‐depleting anti‐CD2 monoclonal antibody
Source: Xenotransplantation. 2023 Nov 13;31(1):e12836. doi: 10.1111/xen.12836 (PMC10909556; doi:10.1111/xen.12836)
Supplement: Supplementary file 6 — Supporting information [file XEN-31-e12836-s001.docx]

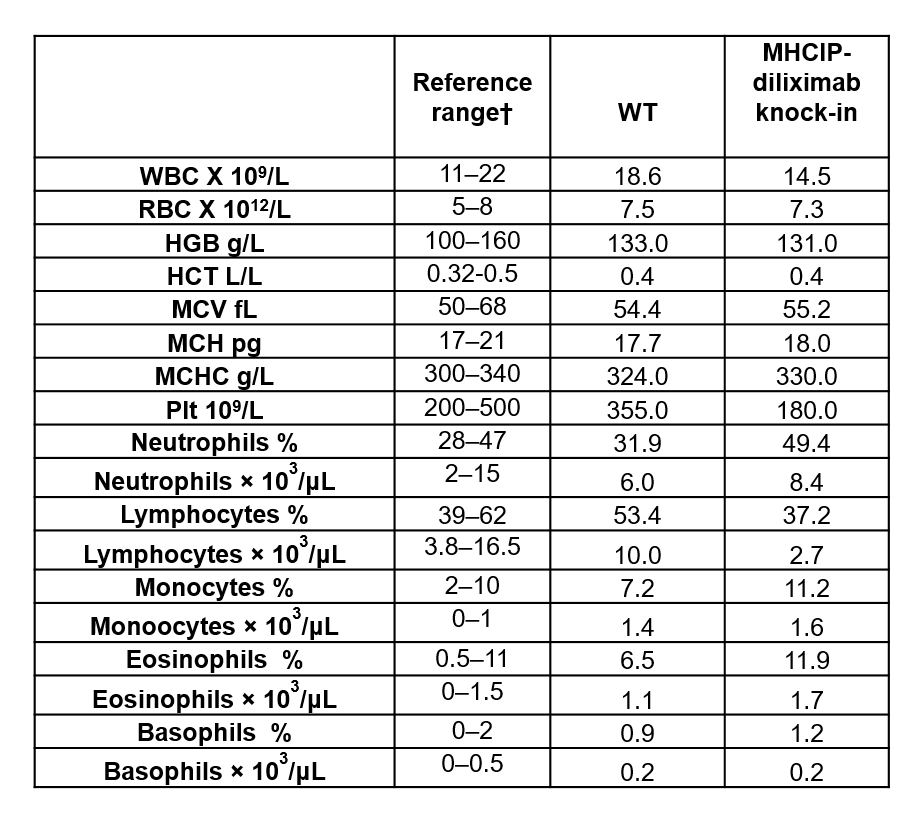


**Supplementary Table 1.** Hematological parameters for representative male WT and MHCIP-diliximab knock-in pigs, demonstrating that the knock-in pig have a normal full blood count. †Reference values were compiled from the following sources: Latimer KS. Duncan & Parse’s Veterinary Laboratory Medicine: Clinical Pathology, 5th ed., Wiley-Blackwell, 2011; Weiss DJ, Wardrop KJ. (2010) Schalm’s Veterinary Hematology, 6th Ed., Wiley-Blackwell and Radostits, O.M., Gay, C.C., Blood, D.C. and Hinchcliff, K.W. (2000) Veterinary Medicine, 9th edn, W.B. Saunders, London, pp. 1819–1822.
